# Decorin is a pivotal effector in the extracellular matrix and tumour microenvironment

**DOI:** 10.18632/oncotarget.23869

**Published:** 2018-01-03

**Authors:** Wen Zhang, Yan Ge, Qian Cheng, Qi Zhang, Lin Fang, Junnian Zheng

**Affiliations:** ^1^ Cancer Institute, Xuzhou Medical University, Xuzhou, China; ^2^ Center of Clinical Oncology, Affiliated Hospital of Xuzhou Medical University, Xuzhou, China; ^3^ Jiangsu Center for The Collaboration and Innovation of Cancer Biotherapy, Cancer Institute, Xuzhou Medical University, Xuzhou, China

**Keywords:** anti-fibrosis, anti-tumour, decorin, pro-inflammatory, proteoglycan

## Abstract

Decorin (DCN), an extracellular matrix (ECM) protein, belongs to the small leucine-rich proteoglycan family. As a pluripotent molecule, DCN regulates the bioactivities of cell growth factors and participates in ECM assembly. Accumulating evidence has shown that DCN acts as a ligand of various cytokines and growth factors by directly or indirectly interacting with the corresponding signalling molecules involved in cell growth, differentiation, proliferation, adhesion and metastasis and that DCN especially plays vital roles in cancer cell proliferation, spread, pro-inflammatory processes and anti-fibrillogenesis. The multifunctional nature of DCN thus enables it to be a potential therapeutic agent for a variety of diseases and shows good prospects for clinical and research applications.

DCN, an extracellular matrix (ECM) protein that belongs to the small leucine-rich proteoglycan family, is widely distributed and plays multifunctional roles in the stroma and epithelial cells. Originally, DCN was known as an effective collagen-binding partner for fibrillogenesis [1] and to modulate key biomechanical parameters of tissue integrity in the tendon, skin and cornea [2]; thus, it was named decorin (DCN). Since being initially cloned in 1986, DCN was discovered to be a structural constituent of the ECM [3]. However, the paradigm has been shifted; it has become increasingly evident that in addition to being a matrix structural protein, DCN affects a wide range of biological processes, including cell growth, differentiation, proliferation, adhesion, spread and migration, and regulates inflammation and fibrillogenesis [4–7]. Two main themes for DCN functions have emerged: maintenance of cellular structure and regulation of signal transduction pathways, culminating in anti-tumourigenic effects. Here, we review the interaction network of DCN and emphasize the biological correlations between these interactions, some of which are expected to be therapeutic intervention targets.

## THE STRUCTURE AND INACTIVATION OF DCN

**Figure 1:**
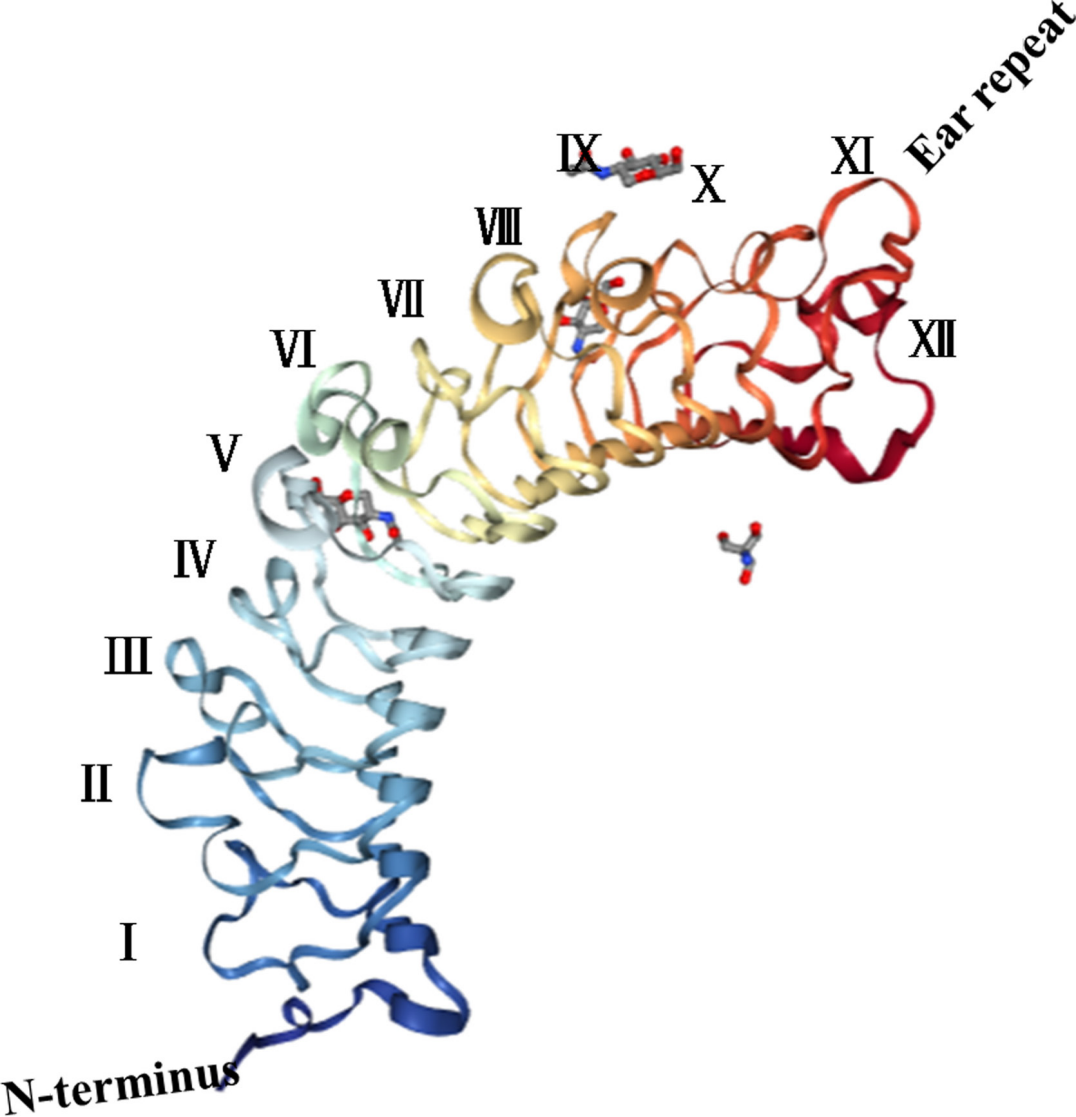
Structure of DCN: Mammalian DCN, is made up of a protein core and a covalently glycosaminoglycan (GAG) chain attached to a serine residue near the N terminus consisting of either chondroitin or dermatan sulfate, leucine-rich repeats (LRRs) altogether 12 LRRs, create a curved solenoid fold, flanked by two cysteine-rich regions, the LRR XI known as the “ear” repeat, its shape looked like a horseshoe or a banana, comprising a convex α-helices and a concave β-sheets formed by leucine-rich repeats

Mammalian DCN, whose complex gene contains 8 exons, is located in chromosome 12q21-q22 and comprises a molecular weight of 92.5-kDa with a coding gene that is of 1080-bp in length. The synthesis and secretion of DCN mainly occur in the rough endoplasmic reticula and Golgi apparatuses of fibroblasts, smooth muscle cells, and macrophages. DCN comprises a 42-kDa conserved protein core that is involved in protein/protein interactions and a single glycosaminoglycan (GAG) chain that is covalently attached to a serine residue near the N terminus consisting of either chondroitin or dermatan sulfate [3, 8]. Structurally, a domain of tandem leucine-rich repeats (LRRs), 12 LRRs in all, form a curved solenoid fold, flanked by two cysteine-rich regions [9] (Figure 1). Brown et al [10] used an X-ray crystal diffraction method to study the crystal structure of DCN and found that its shape resembled a horseshoe or banana, comprising a convex region of α-helices and a concave region of β-sheets formed by leucine-rich repeats that could interact with a variety of protein molecules and that functioned as the structural foundation of the diverse biological functions of DCN. Moreover, each LRR is characterized by unique biological feature that is specific to the known bioactivities of DCN: LRR V/VI assists in DCN binding to VEGFR2 [11]; LRR VII, the collagen-binding sequence is present on the inner surface of the solenoid [12] and directly mediates the interaction between DCN and type I collagen; LRR XII binds to CCN2/CTGF [13]; and LRR XI is known as the “ear” repeat whose truncations or mutations may cause congenital stromal corneal dystrophy [14, 15]. In addition, the GAG (covalently bound glycosaminoglycan) chain highlights a crucial regulatory effect on collagen fibrillogenesis [16], even though it is dispensable in controlling intracellular signalling cascades by cell-surface receptors or signalling molecules. Systematically, depending on the chromosomal composition, amino acid sequence homologies, N-terminal cysteine residues and C-terminal “ear repeats” of the protein core, SLRPs have been divided into three different classes [17]; DCN, together with biglycan (BGN) and aspirin, belongs to the class I SLRPs [18]. Because of the structural similarities between the different SLRPs, they share some of the same biological functions [19]. Morphologically, DCN is a dimer in physiological solutions [10] and is biologically active as a monomer [20]. To date, DCN has gained recognition for its essential roles in regulating the biological processes of inflammatory disorders, fibrotic disorders and cancer, and a numerous applications of DCN as an anticancer therapeutic have been carried out. In recent years, experimental studies have found that several proteases and growth factors can cleave DCN into fragments and inactivate it biologically; matrix metalloproteinases-2 (MMP-2), MMP-3, MMP-7, and membrane type 1-matrix metalloproteinase (MT1-MMP) [21] are the main enzymes involved. Additionally, proteases produced by inflammatory cells can also inactivate DCN [22], through processes known as damage-associated molecular patterns (DAMPs), which can be discerned by pattern recognition receptors (PRRs), such as Toll-like receptors (TLR) 2/4, sparking an inflammatory response [23].

## THE PRIMARY BIOLOGICAL FUNCTIONS OF DCN

### Interaction with growth factor for anti-fibrosis

Fibrosis is a pathological process that occurs in chronic injury, intricate inflammation or long-standing metabolic disease (e.g., diabetes and hypertension) [24, 25] that is characterized by excessive deposition of ECM resulting from an imbalance between ECM synthesis and degradation [26], which occurs in multiple organs, including the lungs [27], heart [28], kidneys [29] and skin [30]. For example, in cases of persistent liver injury, hepatic stellate cells (HSCs), also known as “Ito cells” become activated, and these activated HSCs synthesize and secrete excess ECM that is deposited in the hepatic interstitium, eventually leading to liver fibrosis with frank cirrhosis [31–33].

**Figure 2:**
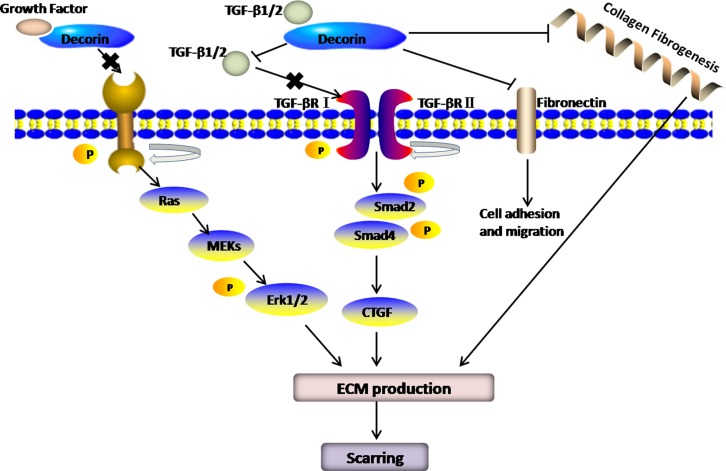
By binding TGF-β and forming inactive TGF-β-DCN complexes, DCN blocks TGF-βRI/II activation and subsequent signalling via Smad2, Smad3 and the ErK1/2 protein to ultimately prevent TGF-β from binding to its receptors, thereby plays a significant role in fibrogenesis In addition, DCN also attenuates TGF-β-mediated fibronectin deposition and collagen fibrogenesis.

In the process of fibrogenesis, transforming growth factor-β (TGF-β) is undoubtedly the most powerful pro-fibrotic cytokines, which can activate fibroblasts, prevent their apoptosis and force them to overproduce matrix components, such as collagen types I, III, and IV and fibronectin [34]. Furthermore, TGF-β also restrains the synthesis of matrix-degrading proteases, and increases the levels of protease inhibitors, such as tissue inhibitor of metalloproteinase1 (TIMP-1) and plasminogen activator inhibitor-1 (PAI-1) [35]. A large number of studies have indicated that DCN is an effective candidate for diminishing TGF-β bioavailability. Through formation of complexes with TGF-β, DCN neutralizes and represses TGF-β, reducing the fibrous scar. Synchronously, by competitive inhibition, DCN impedes the binding of TGF-β to its receptor [36]. Early in 1992, Border et al [6] applied recombinant DCN to ATS-induced glomerulonephritis models and found that DCN could attenuate TGF-β-mediated fibronectin deposition, such the anti-fibrotic effect has observed in many organs [37–39]. Hence, it is plausible that anti-fibrosis via DCN mediated-inactivation of this growth factor may be a valid and logical strategy. With two decades of investigations, the mechanism of the anti-fibrotic properties of DCN has been heavily characterized. By binding to TGF-β and forming inactive TGF-β-DCN complexes, DCN blocks TGF-βRI/II activation and subsequent signalling via Smad2, Smad3 and the ErK1/2 protein [40] (Figure 2) to ultimately prevent TGF-β from binding to its receptors, thereby playing a significant role in fibrogenesis. The isoforms of TGF-β, namely, TGF-β1, 2, and 3, can all bind to the DCN core protein. Additionally, myostatin, another member of the TGF-β superfamily [41], can also be isolated by DCN. Reportedly, exogenous DCN functions by down-regulating TGF-β activity [42].

**Figure 3:**
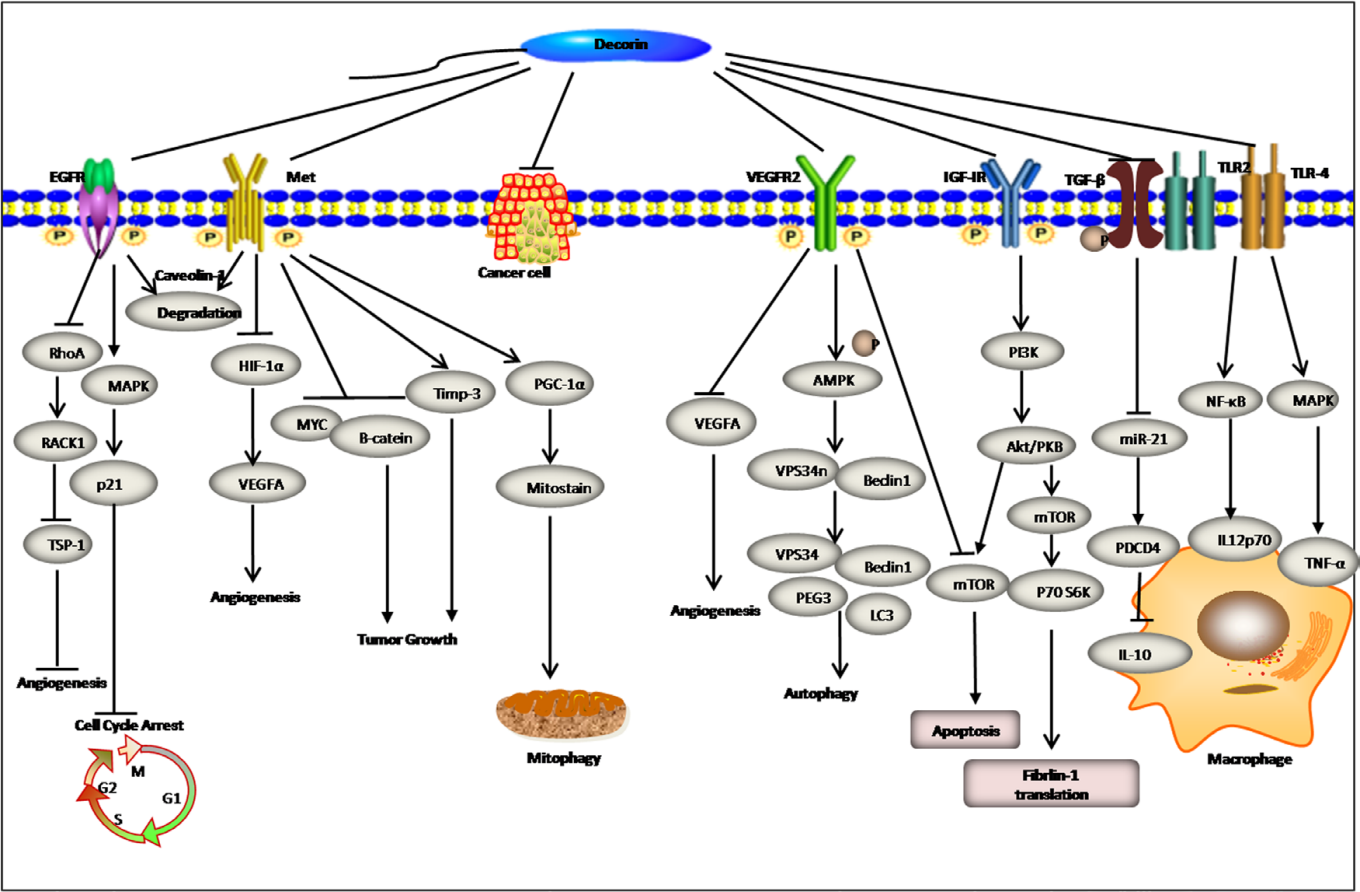
Decorin exhibits multifunction in regulation of inflammation angiogenesis, autophagy, and mitophagy by broad receptor antagonism and attenuation of downstream signaling cascades in tumor cells A detailed description of signalling pathway modulation is provided in the text.

However, the mechanism of anti-fibrosis involves not only the interaction between DCN and TGF-β but also other matrix molecules and cytokines. It has increasingly been accepted that DCN regulates the production of extracellular matrix components (such as fibronectin and thrombospondin-1), inhibits collagen I maturation, and stimulates collagenase. In no-mutation animal models, disruption of the DCN gene leads to skin fragility and abnormal collagen morphology, characterized by uncontrolled lateral fusion of fibrils [43]. More recently, in renal fibroblasts, Schaefer et al [44] has confirmed that by signalling through the phosphorylated PI3K/Akt/mTOR/p70S6K pathway, downstream of IGF-IR, DCN directly augments translation of fibrillin-1 (Figure 3), which has further extended our knowledge of the intricate functions of DCN. In summary, DCN, a potent anti-fibrotic molecule, exerts essential effects towards antagonizing fibrogenesis through a number of distinct mechanisms. Thus, we anticipate DCN becoming an effective therapeutic agent against fibrosis.

### PRO-inflammation and innate immunity

Additionally, as a component of the ECM, soluble SLRPs function as endogenic ligands of Toll-like receptor 2/4 (TLR2/4) and trigger acute inflammatory responses [45] and innate immunity in the case of tissue stress or injury [37, 46–48]. Improved outcomes have been demonstrated in experiments with BGN-deficient mouse models infected with pathogen-regulated or asepsis inflammatory diseases, such as sepsis [47], hydronephrosis [49, 50], systemic lupus nephritis [51], autoimmune perimyocarditis [52] and obesity [53]. Similar to BGN, DCN appears to modulate inflammation through various mechanisms. Primarily, DCN interacts with TLR2/4 on macrophages with a high affinity, concurrently gives rise to transient activation of mitogen-activated protein kinase (MAPK) and NF-κB signal pathways and eventually enhances the release of inflammatory factors such as TNF-α, IL-12p70 and IL-10 [51] (Figure 3).

Furthermore, by down-regulating the bioactivity of TGF-β1, DCN decreases the abundance of oncogenic microRNA (miR-21), a transcriptional inhibitor of a tumour suppressor named programmed cell death protein 4 (PDCD4), in a TLR2/4 independent manner [45] (Figure 3). and ultimately weakens the production of downstream IL-10 (a unique cytokine down-regulated by the pro-inflammatory response in macrophages), thus creating a more pro-inflammatory environment. Ultimately, DCN converts the immune response to a pro-inflammatory state accompanied by growth retardation of tumours. In addition, DCN recruits mononuclear cells to the site of injury by stimulating CCL2 production [54], thus sustaining the inflammatory status. Nevertheless, the mechanism of DCN in regulating the immunoreaction is considerably complex and beyond our knowledge. Daniela et al [55], using a mouse model of Delayed-Type Hypersensitivity, found that DCN could activate receptor tyrosine kinases (RTKs) signal pathways. In turn, this activation induced TNF-α transcription and reduced expression of two adhesion molecules, ICAM-1 and SDC1.

Interestingly, studies in a triple-negative orthotopic breast carcinoma xenograft models, showed an unexpected and paradoxical role for the DCN protein core in inhibiting genes that were necessary for immunomodulatory responses [56]. Therefore, we cannot help but wonder whether DCN initiates pro- or anti-inflammatory responses. Merline et al [45] suggested that only the holonomic DCN, comprising the protein core and GAG chain was capable of triggering a pro-inflammatory response. Accordingly, we boldly speculate that a single protein core can competitively bind to the endogenous ligand of TLR2/4 in the tumour stroma to repress inflammation.

### DCN: an antagonist for tumour growth inhibition

Early genetic studies have demonstrated that deficiency of DCN is permissive for tumour development. These studies tested DCN-null mice with a high-fat diet and showed that these mice have a higher-risk of developing spontaneous intestinal tumours than the control mice; moreover, DCN and p53 double-KO mice showed aggressive T-cell lymphomas with a significantly faster rate of progression than the p53-null mice [57]. Genetically, absence of DCN leads to disorganized intestinal cell maturation, aberrant transformation with suppression of p21 and p27 and elevated β-catenin and Myc [58]. Clinically, lack of DCN expression has been regarded as a strong clinical prognostic biomarker of invasive and metastatic breast cancer [59] and of soft tumours [60]. Via immunohistochemistry and RNA sequencing methods, prominent reduction of matrix constituents including DCN has been detected in the microenvironment of many solid malignancy tissues [61], including breast cancers [59], prostate cancers [62], haemangiomas [63], hepatocellular carcinomas [64], low-high grade urothelial tumours [65], oesophageal squamous cell carcinomas [66], colon cancers [67] and multiple myeloma [68]. Nevertheless, adenoviral delivery of DCN into various solid tumours can counteract tumourigenesis [69–71]. Thus, the mentioned genetic and preclinical discoveries highlight DCN as a promising and viable anti-cancer target for some types of cancer.

#### DCN: an anti-tumour agent via pan-RTK inhibition

Owing to its direct pan-inhibition of numerous key pathways emerging from receptor tyrosine kinase signalling, DCN has been considered as a “guardian from the matrix” [72].

More specifically, monomeric DCN binds to RTKs and evokes receptor dimerization, transient autophosphorylation, caveolin-1-mediated internalization, and, eventually, degradation within lysosomes [73]. EGFR, belonging to the ErbB family, is an important node in the DCN-driven cell signalling pathway and mediates cell adhesion, proliferation, differentiation and apoptosis. Theoretically, the DCN protein is a natural ligand of the EGFR/ErB2 extracellular functional domains and can stimulate rapid formation of receptor dimers and phosphorylation of receptors after DCN/receptor binding. These events trigger a series of signalling cascades and mitogen-activated protein kinase (MAPK) activation and a sudden cytosolic Ca^2+^ increase, with concomitant induction of p21 gene expression, up-regulation of p21 protein (an inhibitor of the cell cycle) and conversion of caspase3 [74] (Figure 3). Collectively, the signalling systems mentioned above paradoxically accelerate cell cycle arrest and induce intrinsic apoptosis, eventually causing tumour cell growth inhibition. Undoubtedly, it is not surprising that DCN also antagonizes other ErbB members, such as ErbB2, ErbB4 and platelet-derived growth factor (PDGF) [59]. Liang et al [75] further reported that overexpression of DCN could block the cell cycle at G1 and decrease the invasive ability of lung cancer A549 cells, further leading to cell apoptosis and inhibition of tumour cell metastasis via decreased phosphorylation of EGFR and increased p53 and p21 expression.

#### DCN and angiogenesis

Angiogenesis is the result of dynamic interactions between a variety of macromolecules in the ECM and cell microenvironment [76]. The role of DCN in neovascularization was discovered from an investigation of the development of cornea. Indeed, DCN not only takes part in angiogenesis, but is also of crucial importance in this process. DCN shows intricate bidirectional regulation of angiogenesis, which can be either pro-angiogenic or anti-angiogenic, depending on the molecular environment.

In a normal and non-tumourigenic microenvironment, such as in the cornea [77], DCN directly supports angiogenesis by boosting endothelial cell attachment to collagen I and α1β2-integrin [78]. During this process, DCN provides the templates of the collagen structure for endothelial cells to induce aggregation of endothelial cells and promotes the formation of blood vessel walls. All of these events can be resistant to proteolytic enzymes, form a firmer fibril network and simultaneously change the biochemical characteristics of the ECM by enhancing its toughness and elasticity [79].

Furthermore, DCN indirectly acts against angiogenesis via interactions with cell surface receptors, signalling molecules and angiogenic growth factors. These macromolecules include epidermal growth factor receptor (EGFR), hepatocyte growth factor (HGF), insulin-like growth factor (IGF), platelet-derived growth factor (PDGF), fibroblast growth factor (FGF), and connective tissue growth factor (CTGF) [80]. Ma et al [81] established animal models of liver fibrosis in BALB/C mice, and found that injections of DCN could accelerate liver regeneration after partial hepatectomy, which may have been related to the angiogenic function of DCN. Likewise, Lai et al [82] reported for the first time that DCN protected endothelia from hyperglycaemia and promoted angiogenesis through IGF-1R/Akt/AP-1/VEGF signalling, which implied that DCN could be a new therapeutic method for patients suffering from DCM (diabetic cardiomyopathy).

In contrast, in the tumour microenvironment, DCN shows an obvious inhibitory function in angiogenesis. When DCN is overexpressed, the tumour growth rate strongly decreases, and tumour angiogenesis is dramatically inhibited. Analyses based on immunohistochemistry, Northern blotting and protein imprinting have revealed that tumour cells expressing DCN show reduced levels of vascular endothelial growth factor [11]. Thus, it is plausible that DCN can inhibit tumour vasifaction. Moreover, by promoting synthesis of matrix metalloproteinase 2 (MMP-2), DCN directly degrades collagen IV in the basement membrane and reduces the proliferation of blood vessels in the body [83]. Meanwhile, as mentioned above, DCN itself can also be cleaved by specific proteases and sequentially transform into a negative regulatory mechanism. Notably, the interactions between DCN and receptor tyrosine kinases (RTKs) are the most widely studied. Met, a receptor tyrosine kinase encoded by the proto-oncogene Met, shows dysregulated expression in numberous malignancies and is involved in cancer growth, metastasis and invasion, which make it a powerful candidate for anti-cancer therapy [84]. Mechanistically, generation of the DCN/Met heterodimer triggers transient autophosphorylation, caveolin-1-mediated internalization, and recruitment of E3 ubiquitin ligase, thus exhibiting an anticancer effect.

Specifically, the newly formed DCN/Met heterodimer continuously degrades the major oncogenes, namely, β-catenin and Myc, via the 26s proteasome after undergoing phosphorylation [85] (Figure 3). Moreover, DCN inhibits hypoxia inducible factor-1α (HIF-1α) and VEGFA via a Met-dependent pathway and induction of the endogenous angiostatin proteins thrombospordin-1 and tissue inhibitor of metallo-proteinases-3 (TIMP3) to promote anti-angiogenesis and prevention of tumour growth and metastasis (Figure 3). Indeed, *in vivo* and *in vitro* studies have strongly suggested that DCN blocks the growth and distant metastasis of breast cancer cells by down-regulating EGFR expression and interfering with the formation of EGFR/ErbB2 dimers [86]. Thus, regulation of angiogenesis is one of the DCN functions in the cellular microenvironment under physiological or pathological stimuli.

#### DCN evokes endothelial cell autophagy and mitophagy

Performing its function as “a guardian from the matrix”, DCN slows the spread and metastasis of tumour cells by indirectly inducing vascular endothelial cell autophagy; the possible mechanisms include endothelial autophagic complex formation and reduced synthesis of autophagy inhibitors. DCN is capable of evoking a prolonged autophagic program in a Peg3 (paternally expressed 3)-dependent manner. Importantly, autophagy functions by targeting the tumour microenvironment rather than acting solely on the actual tumour [56]. Peg3, an imprinted gene commonly silenced in malignant tumours, represents a small subset of DCN-specific inducible genes that are and exclusively modulated within the tumour stroma [87].

In tumorus, autophagy serves as inhibitor of tumour initiation by clearing misfolded proteins, ROS (reactive oxygen species) and other factors [88]. Once stimulated by autophagic stimuli such as starvation and mTOR inhibition, DCN adheres to VEGFR2 on the surfaces of umbilical vein endothelial cells followed by recruitment of Peg3. Subsequently, the pro-autophagic AMPKa/Vps34 signalling axis is activated with concurrent repression of the antiautophagic PI3K/Akt/mTOR/p70S6K signalling pathway. This results in chemotactic attraction of classic autophagy markers, bclin1 and microtubule-associated protein light chain3 (LC3) and formation of the autophagy precursor complex. At the same time, DCN directly prevents the formation of autophagy inhibitor Bel-2 [89] (Figure 3). Goyal et al [90] noted the following: DCN inhibited anti-autophagic signalling by suppressing PI3K/Akt/mTOR/p70S6K activity with concurrent activation of pro-autophagic AMPK-mediated signalling cascades; induced endothelial cell autophagy; reduced the growth of blood vessels in the tumour stroma; and prevented the metastasis and spread of tumour cells. Similar to endothelial autophagy, a novel mechanism has been gradually recognized by investigators whereby DCN functions as a partial agonist of Met for induction of mitochondrial autophagy (Figure 3). This process is named mitophagy. Importantly, this finding further underlines and confirms the tumouricidal role of DCN. Neil et al [91] indicated that DCN evoked tumour cell mitophagy through dynamic co-regulation of PCG-1α and mitostatin via physical interactions between PCG-1α and mitostatin.

Additionally, DCN promotes the expression of FAS/FASL, induces BMP2k (BMP-2 inducible protein kinase) gene expression, and impacts the tumour microenvironment; through attenuation of TGF-β, DCN stabilizes E-cadherin and other proteins in order to inhibit the development and metastasis of tumour cells [92]. Because of its anti-tumour property, DCN has been increasingly applied as an anti-carcinogen to many kinds of malignant tumours. For instance, in pancreatic cancer tissues, DCN can accelerate the degradation of the ECM surrounding the cancer cell to increase infiltration of chemotherapeutic agents into the tumour lesion, thus improving the cure effect of the agent for the pancreatic cancer [93]. Goldoni et al [86] found that DCN could effectively inhibit the growth and distant metastasis of breast cancer cells in primary lesion, thus they hypothesized that DCN could be used as a candidate drugs in the targeted therapy of breast cancer. It is noteworthy that the anti-tumour role of DCN is not only limited to local tumours but also involved in haematogenous tumours, and that inhibition of cell growth by DCN shows tumour cell selectivity [94].

## VIRUS-MEDIATED DECORIN FOR CANCER TREATMENT

Tumour gene-virus treatment [95], the basic idea of which involves integration of anti-oncogene into an oncolytic adenovirus (OAd), combines virus therapy with gene therapy for cancer inhibition. The OAd, which is transformed with genetic engineering technology, kills tumour cells through selective replication of the virus without toxicity towards normal cells and has become a promising vector [96]. Reed et al [97] have provided evidence that DCN gene therapy could repress tumours growth and that DCN gene therapy represented an independent or adjunctive therapeutic modality against cancer. Similarly, Xu et al [71] constructed Ad-DCN and found that recombinant DCN could inhibit Met and the Wnt/β-catenin signalling axis and prevent bone metastasis of prostate cancer cells.

Recent evidences have shown that, for solid tumors, connective tissue and ECM may have a prominent role in inhibiting viral spread after their administration [98], and the tumors of patients in the clinical trials were composed of heterogeneous tumor cell populations that actively recruited or produced immunosuppressive factors, generating a highly immunosuppressive tumor microenvironment [99]. Since DCN regulated the production and assembly of the ECM at several levels, and remodeled connective tissues to overcome the extracellular matrix barrier, Choi et al [100] reported that intratumoural injection of Ad-ΔE1B-DCN not only decreased the ECM components in tumour tissues but also reduced B16BL6 melanoma cell pulmonary metastases.

Moreover, DCN acts as an adjuvant for overcoming TGF-β-mediated immunosuppression. Yun et al [101] aimed to heighten the anti-tumour effect by designing and generating a novel oncolytic adenovirus (Ad) that co-expressed Interleukin (IL)-12 and DCN (RdB/IL12/DCN), which is a potent anti-tumour cytokine. There were significantly higher levels of expression of immune-modulation genes, such as interferon (IFN)-γ, tumour necrosis factor-α, and monocyte chemoattractant protein-1. Meanwhile, RdB/IL-12/DCN attenuated intratumoural TGF-β expression, increased infiltration of CD8^+^ T cells and proficient viral spreading within tumour tissues. This therapeutic mechanism of a cytokine plus DCN is a promising cancer immunotherapeutic approach for overcoming tumour-induced immunosuppression.

## CONCLUSION AND OUTLOOK

DCN, a prototypical small leucine-rich proteoglycan, was initially defined as structural constituent of the ECM and as a participant in fibrillogenesis. Soon afterwards, it was demonstrated that DCN was essential for anti-fibrosis and pro-inflammatory processes. Furthermore, emerging studies have suggested that DCN is a pan-RTK inhibitor that possesses anti-tumour capabilities involved in cell growth, differentiation, survival and metastasis. Therefore, DCN is promising therapeutic agent for a variety of diseases and shows good prospects for clinical applications as an anti-tumour therapy. In recent years, a recombinant therapeutic approach with DCN and an oncolytic adenovirus has made significant progress in animal models. However, as part of the clinical application process, many problems need to be addressed by researchers, including the reduced ability of viral replication in the hypoxic microenvironment of the solid tumour and the limited distribution of the virus. Furthermore, extensive preclinical studies are needed because recombinant DCN has not met the standard of clinical drugs. In the future, our research will focus on solving these problems and lead to new diagnostic and individualized treatment approaches for cancers.

## References

[1] Chen S, Young MF, Chakravarti S, Birk DE (2014). Interclass small leucine-rich repeat proteoglycan interactions regulate collagen fibrillogenesis and corneal stromal assembly. Matrix Biol.

[2] Robinson PS, Huang TF, Kazam E, Iozzo RV, Birk DE, Soslowsky LJ (2005). Influence of decorin and biglycan on mechanical properties of multiple tendons in knockout mice. J Biomech Eng.

[3] Krusius T, Ruoslahti E (1986). Primary structure of an extracellular matrix proteoglycan core protein deduced from cloned cDNA. Proc Natl Acad Sci U S A.

[4] Ruoslahti E, Yamaguchi Y (1991). Proteoglycans as modulators of growth factor activities. Cell.

[5] Yamaguchi Y, Ruoslahti E (1988). Expression of human proteoglycan in Chinese hamster ovary cells inhibits cell proliferation. Nature.

[6] Border WA, Noble NA, Yamamoto T, Harper JR, Yamaguchi Y, Pierschbacher MD, Ruoslahti E (1992). Natural inhibitor of transforming growth factor-beta protects against scarring in experimental kidney disease. Nature.

[7] Yamaguchi Y, Mann DM, Ruoslahti E (1990). Negative regulation of transforming growth factor-beta by the proteoglycan decorin. Nature.

[8] Mann DM, Yamaguchi Y, Bourdon MA, Ruoslahti E (1990). Analysis of glycosaminoglycan substitution in decorin by site-directed mutagenesis. J Biol Chem.

[9] Scott PG, McEwan PA, Dodd CM, Bergmann EM, Bishop PN, Bella J (2004). Crystal structure of the dimeric protein core of decorin, the archetypal small leucine-rich repeat proteoglycan. Proc Natl Acad Sci U S A.

[10] Brown CT, Lin P, Walsh MT, Gantz D, Nugent MA, Trinkaus-Randall V (2002). Extraction and purification of decorin from corneal stroma retain structure and biological activity. Protein Expr Purif.

[11] Khan GA, Girish GV, Lala N, Di Guglielmo GM, Lala PK (2011). Decorin is a novel VEGFR-2-binding antagonist for the human extravillous trophoblast. Mol Endocrinol.

[12] Kalamajski S, Aspberg A, Oldberg A (2007). The decorin sequence SYIRIADTNIT binds collagen type I. J Biol Chem.

[13] Vial C, Gutierrez J, Santander C, Cabrera D, Brandan E (2011). Decorin interacts with connective tissue growth factor (CTGF)/CCN2 by LRR12 inhibiting its biological activity. J Biol Chem.

[14] Chen S, Birk DE (2013). The regulatory roles of small leucine-rich proteoglycans in extracellular matrix assembly. FEBS J.

[15] Chen S, Sun M, Meng X, Iozzo RV, Kao WW, Birk DE (2011). Pathophysiological mechanisms of autosomal dominant congenital stromal corneal dystrophy: C-terminal-truncated decorin results in abnormal matrix assembly and altered expression of small leucine-rich proteoglycans. Am J Pathol.

[16] Iozzo RV, Schaefer L (2015). Proteoglycan form and function: a comprehensive nomenclature of proteoglycans. Matrix Biol.

[17] McEwan PA, Scott PG, Bishop PN, Bella J (2006). Structural correlations in the family of small leucine-rich repeat proteins and proteoglycans. J Struct Biol.

[18] Lorenzo P, Aspberg A, Onnerfjord P, Bayliss MT, Neame PJ, Heinegard D (2001). Identification and characterization of asporin. a novel member of the leucine-rich repeat protein family closely related to decorin and biglycan. J Biol Chem.

[19] Corsi A, Xu T, Chen XD, Boyde A, Liang J, Mankani M, Sommer B, Iozzo RV, Eichstetter I, Robey PG, Bianco P, Young MF (2002). Phenotypic effects of biglycan deficiency are linked to collagen fibril abnormalities, are synergized by decorin deficiency, and mimic Ehlers-Danlos-like changes in bone and other connective tissues. J Bone Miner Res.

[20] Islam M, Gor J, Perkins SJ, Ishikawa Y, Bachinger HP, Hohenester E (2013). The concave face of decorin mediates reversible dimerization and collagen binding. J Biol Chem.

[21] Mimura T, Han KY, Onguchi T, Chang JH, Kim TI, Kojima T, Zhou Z, Azar DT (2009). MT1-MMP-mediated cleavage of decorin in corneal angiogenesis. J Vasc Res.

[22] Parkinson LG, Toro A, Zhao H, Brown K, Tebbutt SJ, Granville DJ (2015). Granzyme B mediates both direct and indirect cleavage of extracellular matrix in skin after chronic low-dose ultraviolet light irradiation. Aging Cell.

[23] Schaefer L (2014). Complexity of danger: the diverse nature of damage-associated molecular patterns. J Biol Chem.

[24] Chan EC, Jiang F, Peshavariya HM, Dusting GJ (2009). Regulation of cell proliferation by NADPH oxidase-mediated signaling: potential roles in tissue repair, regenerative medicine and tissue engineering. Pharmacol Ther.

[25] Rosenbloom J, Castro SV, Jimenez SA (2010). Narrative review: fibrotic diseases: cellular and molecular mechanisms and novel therapies. Ann Intern Med.

[26] Schnaper HW (1995). Balance between matrix synthesis and degradation: a determinant of glomerulosclerosis. Pediatr Nephrol.

[27] Strieter RM (2008). What differentiates normal lung repair and fibrosis? Inflammation, resolution of repair, and fibrosis. Proc Am Thorac Soc.

[28] Kanisicak O, Khalil H, Ivey MJ, Karch J, Maliken BD, Correll RN, Brody MJ, Lin SCJ, Aronow BJ, Tallquist MD, Molkentin JD (2016). Genetic lineage tracing defines myofibroblast origin and function in the injured heart. Nat Commun.

[29] Mack M, Yanagita M (2015). Origin of myofibroblasts and cellular events triggering fibrosis. Kidney Int.

[30] Hellstrom M, Hellstrom S, Engstrom-Laurent A, Bertheim U (2014). The structure of the basement membrane zone differs between keloids, hypertrophic scars and normal skin: a possible background to an impaired function. J Plast Reconstr Aesthet Surg.

[31] Gressner AM (1996). Transdifferentiation of hepatic stellate cells (Ito cells) to myofibroblasts: a key event in hepatic fibrogenesis. Kidney Int Suppl.

[32] Shek FW, Benyon RC (2004). How can transforming growth factor beta be targeted usefully to combat liver fibrosis?. Eur J Gastroenterol Hepatol.

[33] Knittel T, Kobold D, Saile B, Grundmann A, Neubauer K, Piscaglia F, Ramadori G (1999). Rat liver myofibroblasts and hepatic stellate cells: different cell populations of the fibroblast lineage with fibrogenic potential. Gastroenterology.

[34] Kanzler S, Lohse AW, Keil A, Henninger J, Dienes HP, Schirmacher P, Rose-John S, zum Buschenfelde KH, Blessing M (1999). TGF-beta1 in liver fibrosis: an inducible transgenic mouse model to study liver fibrogenesis. Am J Physiol.

[35] Dudas J, Kovalszky I, Gallai M, Nagy JO, Schaff Z, Knittel T, Mehde M, Neubauer K, Szalay F, Ramadori G (2001). Expression of decorin, transforming growth factor-beta 1, tissue inhibitor metalloproteinase 1 and 2, and type IV collagenases in chronic hepatitis. Am J Clin Pathol.

[36] Harper JR, Spiro RC, Gaarde WA, Tamura RN, Pierschbacher MD, Noble NA, Stecker KK, Border WA (1994). Role of transforming growth factor beta and decorin in controlling fibrosis. Methods Enzymol.

[37] Al Haj Zen A, Caligiuri G, Sainz J, Lemitre M, Demerens C, Lafont A (2006). Decorin overexpression reduces atherosclerosis development in apolipoprotein E-deficient mice. Atherosclerosis.

[38] Kolb M, Margetts PJ, Sime PJ, Gauldie J (2001). Proteoglycans decorin and biglycan differentially modulate TGF-beta-mediated fibrotic responses in the lung. Am J Physiol Lung Cell Mol Physiol.

[39] Shi YF, Zhang Q, Cheung PY, Shi L, Fong CC, Zhang Y, Tzang CH, Chan BP, Fong WF, Chun J, Kung HF, Yang M (2006). Effects of rhDecorin on TGF-beta1 induced human hepatic stellate cells LX-2 activation. Biochim Biophys Acta.

[40] Baghy K, Iozzo RV, Kovalszky I (2012). Decorin-TGFbeta axis in hepatic fibrosis and cirrhosis. J Histochem Cytochem.

[41] Jarvinen TA, Prince S (2015). Decorin: a growth factor antagonist for tumor growth inhibition. Biomed Res Int.

[42] Zhu J, Li Y, Shen W, Qiao C, Ambrosio F, Lavasani M, Nozaki M, Branca MF, Huard J (2007). Relationships between transforming growth factor-beta1, myostatin, and decorin: implications for skeletal muscle fibrosis. J Biol Chem.

[43] Jarvinen TA, Ruoslahti E (2010). Target-seeking antifibrotic compound enhances wound healing and suppresses scar formation in mice. Proc Natl Acad Sci U S A.

[44] Schaefer L, Tsalastra W, Babelova A, Baliova M, Minnerup J, Sorokin L, Grone HJ, Reinhardt DP, Pfeilschifter J, Iozzo RV, Schaefer RM (2007). Decorin-mediated regulation of fibrillin-1 in the kidney involves the insulin-like growth factor-I receptor and Mammalian target of rapamycin. Am J Pathol.

[45] Merline R, Moreth K, Beckmann J, Nastase MV, Zeng-Brouwers J, Tralhao JG, Lemarchand P, Pfeilschifter J, Schaefer RM, Iozzo RV, Schaefer L (2011). Signaling by the matrix proteoglycan decorin controls inflammation and cancer through PDCD4 and MicroRNA-21. Sci Signal.

[46] Schaefer L, Macakova K, Raslik I, Micegova M, Grone HJ, Schonherr E, Robenek H, Echtermeyer FG, Grassel S, Bruckner P, Schaefer RM, Iozzo RV, Kresse H (2002). Absence of decorin adversely influences tubulointerstitial fibrosis of the obstructed kidney by enhanced apoptosis and increased inflammatory reaction. Am J Pathol.

[47] Schaefer L, Babelova A, Kiss E, Hausser HJ, Baliova M, Krzyzankova M, Marsche G, Young MF, Mihalik D, Gotte M, Malle E, Schaefer RM, Grone HJ (2005). The matrix component biglycan is proinflammatory and signals through Toll-like receptors 4 and 2 in macrophages. J Clin Invest.

[48] Vij N, Roberts L, Joyce S, Chakravarti S (2005). Lumican regulates corneal inflammatory responses by modulating Fas-Fas ligand signaling. Invest Ophthalmol Vis Sci.

[49] Schaefer L (2011). Small leucine-rich proteoglycans in kidney disease. J Am Soc Nephrol.

[50] Babelova A, Moreth K, Tsalastra-Greul W, Zeng-Brouwers J, Eickelberg O, Young MF, Bruckner P, Pfeilschifter J, Schaefer RM, Grone HJ, Schaefer L (2009). Biglycan, a danger signal that activates the NLRP3 inflammasome via toll-like and P2X receptors. J Biol Chem.

[51] Moreth K, Brodbeck R, Babelova A, Gretz N, Spieker T, Zeng-Brouwers J, Pfeilschifter J, Young MF, Schaefer RM, Schaefer L (2010). The proteoglycan biglycan regulates expression of the B cell chemoattractant CXCL13 and aggravates murine lupus nephritis. J Clin Invest.

[52] Popovic ZV, Wang S, Papatriantafyllou M, Kaya Z, Porubsky S, Meisner M, Bonrouhi M, Burgdorf S, Young MF, Schaefer L, Grone HJ (2011). The proteoglycan biglycan enhances antigen-specific T cell activation potentially via MyD88 and TRIF pathways and triggers autoimmune perimyocarditis. J Immunol.

[53] Adapala VJ, Ward M, Ajuwon KM (2012). Adipose tissue biglycan as a potential anti-inflammatory target of sodium salicylate in mice fed a high fat diet. J Inflamm (Lond).

[54] Koninger J, Giese NA, Bartel M, di Mola FF, Berberat PO, di Sebastiano P, Giese T, Buchler MW, Friess H (2006). The ECM proteoglycan decorin links desmoplasia and inflammation in chronic pancreatitis. J Clin Pathol.

[55] Seidler DG, Mohamed NA, Bocian C, Stadtmann A, Hermann S, Schafers K, Schafers M, Iozzo RV, Zarbock A, Gotte M (2011). The role for decorin in delayed-type hypersensitivity. J Immunol.

[56] Buraschi S, Neill T, Owens RT, Iniguez LA, Purkins G, Vadigepalli R, Evans B, Schaefer L, Peiper SC, Wang ZX, Iozzo RV (2012). Decorin protein core affects the global gene expression profile of the tumor microenvironment in a triple-negative orthotopic breast carcinoma xenograft model. PLoS One.

[57] Iozzo RV, Chakrani F, Perrotti D, McQuillan DJ, Skorski T, Calabretta B, Eichstetter I (1999). Cooperative action of germ-line mutations in decorin and p53 accelerates lymphoma tumorigenesis. Proc Natl Acad Sci U S A.

[58] Bi X, Tong C, Dockendorff A, Bancroft L, Gallagher L, Guzman G, Iozzo RV, Augenlicht LH, Yang W (2008). Genetic deficiency of decorin causes intestinal tumor formation through disruption of intestinal cell maturation. Carcinogenesis.

[59] Goldoni S, Iozzo RV (2008). Tumor microenvironment: modulation by decorin and related molecules harboring leucine-rich tandem motifs. Int J Cancer.

[60] Matsumine A, Shintani K, Kusuzaki K, Matsubara T, Satonaka H, Wakabayashi T, Iino T, Uchida A (2007). Expression of decorin, a small leucine-rich proteoglycan, as a prognostic factor in soft tissue tumors. J Surg Oncol.

[61] Iozzo RV, Buraschi S, Genua M, Xu SQ, Solomides CC, Peiper SC, Gomella LG, Owens RC, Morrione A (2011). Decorin antagonizes IGF receptor I (IGF-IR) function by interfering with IGF-IR activity and attenuating downstream signaling. J Biol Chem.

[62] Henke A, Grace OC, Ashley GR, Stewart GD, Riddick AC, Yeun H, O'Donnell M, Anderson RA, Thomson AA (2012). Stromal expression of decorin, Semaphorin6D, SPARC, Sprouty1 and Tsukushi in developing prostate and decreased levels of decorin in prostate cancer. PLoS One.

[63] Salomaki HH, Sainio AO, Soderstrom M, Pakkanen S, Laine J, Jarvelainen HT (2008). Differential expression of decorin by human malignant and benign vascular tumors. J Histochem Cytochem.

[64] Duncan MB (2013). Extracellular matrix transcriptome dynamics in hepatocellular carcinoma. Matrix Biol.

[65] Sainio A, Nyman M, Lund R, Vuorikoski S, Bostrom P, Laato M, Bostrom PJ, Jarvelainen H (2013). Lack of decorin expression by human bladder cancer cells offers new tools in the therapy of urothelial malignancies. PLoS One.

[66] Wu IC, Wu DC, Huang CC, Lin HS, Chen YK, Tsai HJ, Lu CY, Chou SH, Chou YP, Li LH, Tai SY, Wu MT (2010). Plasma decorin predicts the presence of esophageal squamous cell carcinoma. Int J Cancer.

[67] Nyman MC, Sainio AO, Pennanen MM, Lund RJ, Vuorikoski S, Sundstrom JT, Jarvelainen HT (2015). Decorin in human colon cancer: localization in vivo and effect on cancer cell behavior in vitro. J Histochem Cytochem.

[68] Kristensen IB, Pedersen L, Ro TB, Christensen JH, Lyng MB, Rasmussen LM, Ditzel HJ, Borset M, Abildgaard N (2013). Decorin is down-regulated in multiple myeloma and MGUS bone marrow plasma and inhibits HGF-induced myeloma plasma cell viability and migration. Eur J Haematol.

[69] Jiang G, Sun C, Li RH, Wei ZP, Zheng JN, Liu YQ (2015). Enhanced antitumor efficacy of a novel oncolytic adenovirus combined with temozolomide in the treatment of melanoma in vivo. J Cancer Res Clin Oncol.

[70] Kaliberova LN, Krendelchtchikova V, Harmon DK, Stockard CR, Petersen AS, Markert JM, Gillespie GY, Grizzle WE, Buchsbaum DJ, Kaliberov SA (2009). CRAdRGDflt-IL24 virotherapy in combination with chemotherapy of experimental glioma. Cancer Gene Ther.

[71] Xu W, Neill T, Yang Y, Hu Z, Cleveland E, Wu Y, Hutten R, Xiao X, Stock SR, Shevrin D, Kaul K, Brendler C, Iozzo RV, Seth P (2015). The systemic delivery of an oncolytic adenovirus expressing decorin inhibits bone metastasis in a mouse model of human prostate cancer. Gene Ther.

[72] Neill T, Schaefer L, Iozzo RV (2012). Decorin: a guardian from the matrix. Am J Pathol.

[73] Zhu JX, Goldoni S, Bix G, Owens RT, McQuillan DJ, Reed CC, Iozzo RV (2005). Decorin evokes protracted internalization and degradation of the epidermal growth factor receptor via caveolar endocytosis. J Biol Chem.

[74] Seidler DG, Goldoni S, Agnew C, Cardi C, Thakur ML, Owens RT, McQuillan DJ, Iozzo RV (2006). Decorin protein core inhibits in vivo cancer growth and metabolism by hindering epidermal growth factor receptor function and triggering apoptosis via caspase-3 activation. J Biol Chem.

[75] Liang S, Xu JF, Cao WJ, Li HP, Hu CP (2013). Human decorin regulates proliferation and migration of human lung cancer A549 cells. Chin Med J (Engl).

[76] Stapor P, Wang X, Goveia J, Moens S, Carmeliet P (2014). Angiogenesis revisited - role and therapeutic potential of targeting endothelial metabolism. J Cell Sci.

[77] Mohan RR, Tovey JC, Sharma A, Schultz GS, Cowden JW, Tandon A (2011). Targeted decorin gene therapy delivered with adeno-associated virus effectively retards corneal neovascularization in vivo. PLoS One.

[78] Santra M, Santra S, Zhang J, Chopp M (2008). Ectopic decorin expression up-regulates VEGF expression in mouse cerebral endothelial cells via activation of the transcription factors Sp1, HIF1alpha, and Stat3. J Neurochem.

[79] Reese SP, Underwood CJ, Weiss JA (2013). Effects of decorin proteoglycan on fibrillogenesis, ultrastructure, and mechanics of type I collagen gels. Matrix Biol.

[80] Jarvelainen H, Sainio A, Wight TN (2015). Pivotal role for decorin in angiogenesis. Matrix Biol.

[81] Ma R, Chen J, Li Z, Tang J, Wang Y, Cai X (2014). Decorin accelerates the liver regeneration after partial hepatectomy in fibrotic mice. Chin Med J (Engl).

[82] Lai J, Chen F, Chen J, Ruan G, He M, Chen C, Tang J, Wang DW (2017). Overexpression of decorin promoted angiogenesis in diabetic cardiomyopathy via IGF1R-AKT-VEGF signaling. Sci Rep.

[83] Al Haj Zen A, Lafont A, Durand E, Brasselet C, Lemarchand P, Godeau G, Gogly B (2003). Effect of adenovirus-mediated overexpression of decorin on metalloproteinases, tissue inhibitors of metalloproteinases and cytokines secretion by human gingival fibroblasts. Matrix Biol.

[84] Zhang J, Jiang X, Jiang Y, Guo M, Zhang S, Li J, He J, Liu J, Wang J, Ouyang L (2016). Recent advances in the development of dual VEGFR and c-Met small molecule inhibitors as anticancer drugs. Eur J Med Chem.

[85] Buraschi S, Pal N, Tyler-Rubinstein N, Owens RT, Neill T, Iozzo RV (2010). Decorin antagonizes Met receptor activity and down-regulates {beta}-catenin and Myc levels. J Biol Chem.

[86] Goldoni S, Seidler DG, Heath J, Fassan M, Baffa R, Thakur ML, Owens RT, McQuillan DJ, Iozzo RV (2008). An antimetastatic role for decorin in breast cancer. Am J Pathol.

[87] Nye MD, Hoyo C, Huang Z, Vidal AC, Wang F, Overcash F, Smith JS, Vasquez B, Hernandez B, Swai B, Oneko O, Mlay P, Obure J (2013). Associations between methylation of paternally expressed gene 3 (PEG3), cervical intraepithelial neoplasia and invasive cervical cancer. PLoS One.

[88] Torres A, Gubbiotti MA, Iozzo RV (2017). Decorin-inducible Peg3 evokes beclin 1-mediated autophagy and thrombospondin 1-mediated angiostasis. J Biol Chem.

[89] Champagne FA, Curley JP, Swaney WT, Hasen NS, Keverne EB (2009). Paternal influence on female behavior: the role of Peg3 in exploration, olfaction, and neuroendocrine regulation of maternal behavior of female mice. Behav Neurosci.

[90] Goyal A, Neill T, Owens RT, Schaefer L, Iozzo RV (2014). Decorin activates AMPK, an energy sensor kinase, to induce autophagy in endothelial cells. Matrix Biol.

[91] Neill T, Torres A, Buraschi S, Owens RT, Hoek JB, Baffa R, Iozzo RV (2014). Decorin induces mitophagy in breast carcinoma cells via peroxisome proliferator-activated receptor gamma coactivator-1alpha (PGC-1alpha) and mitostatin. J Biol Chem.

[92] Neill T, Schaefer L, Iozzo RV (2016). Decorin as a multivalent therapeutic agent against cancer. Adv Drug Deliv Rev.

[93] Na Y, Choi JW, Kasala D, Hong J, Oh E, Li Y, Jung SJ, Kim SW, Yun CO (2015). Potent antitumor effect of neurotensin receptor-targeted oncolytic adenovirus co-expressing decorin and Wnt antagonist in an orthotopic pancreatic tumor model. J Control Release.

[94] Tralhao JG, Schaefer L, Micegova M, Evaristo C, Schonherr E, Kayal S, Veiga-Fernandes H, Danel C, Iozzo RV, Kresse H, Lemarchand P (2003). In vivo selective and distant killing of cancer cells using adenovirus-mediated decorin gene transfer. FASEB J.

[95] Alemany R, Gomez-Manzano C, Balague C, Yung WK, Curiel DT, Kyritsis AP, Fueyo J (1999). Gene therapy for gliomas: molecular targets, adenoviral vectors, and oncolytic adenoviruses. Exp Cell Res.

[96] Cerullo V, Vaha-Koskela M, Hemminki A (2012). Oncolytic adenoviruses: a potent form of tumor immunovirotherapy. Oncoimmunology.

[97] Reed CC, Gauldie J, Iozzo RV (2002). Suppression of tumorigenicity by adenovirus-mediated gene transfer of decorin. Oncogene.

[98] Parato KA, Senger D, Forsyth PA, Bell JC (2005). Recent progress in the battle between oncolytic viruses and tumours. Nat Rev Cancer.

[99] Zwirner NW, Croci DO, Domaica CI, Rabinovich GA (2010). Overcoming the hurdles of tumor immunity by targeting regulatory pathways in innate and adaptive immune cells. Curr Pharm Des.

[100] Choi IK, Lee YS, Yoo JY, Yoon AR, Kim H, Kim DS, Seidler DG, Kim JH, Yun CO (2010). Effect of decorin on overcoming the extracellular matrix barrier for oncolytic virotherapy. Gene Ther.

[101] Oh E, Choi IK, Hong J, Yun CO (2017). Oncolytic adenovirus coexpressing interleukin-12 and decorin overcomes Treg-mediated immunosuppression inducing potent antitumor effects in a weakly immunogenic tumor model. Oncotarget.

